# Development of *Sarcophaga princeps* Wiedemann (Diptera: Sarcophagidae) Under Constant Temperature and Its Implication in Forensic Entomology

**DOI:** 10.3390/insects16111153

**Published:** 2025-11-11

**Authors:** Liangliang Li, Yingna Zhang, Gengwang Hu, Yumeng Zhuo, Jianjun Jin, Qiang Fang, Xuebo Li, Shujin Li, Yu Wang

**Affiliations:** 1 Characteristic Laboratory of Forensic Science in the Universities of Shandong Province, Shandong University of Political Science and Law, Jinan 250014, China; lill@sdupsl.edu.cn (L.L.); 900338@sdupsl.edu.cn (Y.Z.); 900336@sdupsl.edu.cn (J.J.); 900272@sdupsl.edu.cn (Q.F.);; 2 Hebei Key Laboratory of Forensic Medicine, Shijiazhuang 050017, China; 3 Department of Anatomy, Shihezi University School of Medicine, Shihezi 832003, China; zhangyingna_oliver@shzu.edu.cn; 4 Department of Forensic Medicine, Soochow University, Suzhou 215000, China; 20214221010@stu.suda.edu.cn

**Keywords:** forensic entomology, postmortem interval, sarcophagid, development

## Abstract

Sarcophagidae are often the first sarcosaprophagous insects to colonize corpses in specialized cases such as indoor discoveries and burials, making them forensically crucial for estimating the minimum postmortem interval (PMI_min_). Among these, *Sarcophaga princeps* Wiedemann (Diptera: Sarcophagidae) is a prominent species frequently associated with both buried and indoor bodies. In this study, the development time of *S. princeps* from larvae to adults at constant temperatures of 16, 19, 22, 25, 28, 31, and 34 °C was studied, and the times required were 1090.00 ± 57.65, 721.00 ± 8.72, 562.33 ± 27.21, 416.67 ± 27.70, 356.33 ± 16.01, 327.00 ± 7.94, and 313.67 ± 5.69 h, respectively, demonstrating a significant inverse relationship with temperature. Various developmental models were constructed using the basic developmental data, including the isomorphen diagram, isomegalen diagram, nonlinear thermodynamic Optim SSI model, and logistic regression model. These models enable the estimation of the developmental age of the specimens. In addition, the lower critical thermal threshold (*T_L_*), intrinsic optimum temperature (*T_Φ_*), and upper critical thermal threshold (*T_H_*) estimated by the nonlinear thermodynamic Optim SSI model were 11.11 °C, 21.85 °C, and 35.88 °C. This study provides comprehensive developmental data of *S. princeps* for PMI_min_ estimation.

## Simple Summary

Sarcophagidae are often the first insects to arrive and larviposit at corpses. They are forensically important insect species for estimating minimum postmortem intervals (PMI_min_). *Sarcophaga princeps* Wiedemann (Diptera: Sarcophagidae) is an important species of sarcosaprophagous insects which colonizes buried and indoor bodies. This study was conducted to provide developmental data of *S. princeps* for PMI_min_ estimation. The development of *S. princeps* from larvae to adults was studied at constant temperatures ranging from 16 to 34 °C. Developmental times decrease from 1090.00 ± 57.65 to 313.67 ± 5.69 h as temperatures increase. Developmental models were constructed that can be used to estimate the age of this species. In addition, the lower critical thermal threshold (*T_L_*), intrinsic optimum temperature (*T_Φ_*), and upper critical thermal threshold (*T_H_*) were estimated at 11.11 °C, 21.85 °C, and 35.88 °C using the Optim SSI model. This study provides baseline data for postmortem PMI_min_ in forensic entomology.

## 1. Introduction

Accurate estimation of postmortem interval (PMI) is of particular importance for forensic investigations [1,2]. The development time of the offspring of sarcosaprophagous insects represents the minimum time since death. Estimating this minimum postmortem interval (PMI_min_) in forensic entomology depends on precise developmental data of sarcosaprophagous insects [3]. Consequently, the growth and development patterns of insects collected from corpses are a focal point of forensic entomological research [4,5].

Flesh flies (Diptera: Sarcophagidae) are among the first insects to arrive at a corpse, being similar to blow flies (Diptera: Calliphoridae) [6,7,8]. Sarcophagids exhibit an ovoviviparous reproductive strategy, which may enable their offspring to develop more rapidly compared to the more extensively studied calliphorid species [9]. Sarcophagid larvae are generally larger than calliphorid larvae of the same age, a characteristic that facilitates their collection at crime scenes [6]. Recent studies suggest that sarcophagids may be the PMI_min_ indicator insect at crime scenes [10,11], especially for corpses that are in indoor and burial environments [12]. However, there are few studies on Sarcophagidae development, which limits the application of Sarcophagidae in PMI_min_ estimation [6,13].

*Sarcophaga princeps* Wiedemann, 1830 (syn. *Seniorwhitea reciproca* Walker, 1856), is distributed across the Oriental region and portions of the Palearctic region [14,15,16]. It is commonly found on dead fish, animals, and other decaying organic matter [14,15],. and can colonize decomposing bodies [17,18]. This species was the dominant species found on carcasses in our buried carcass experiments (unpublished data). This dominance is likely because the larvae are directly larviposited by this species and can reach carcasses buried beneath the soil via crawling, whereas other species (e.g., *Chrysomya megacephala*) are prevented from colonizing the carcasses due to soil isolation [19]. Burial is one of the most common means by which criminals conceal evidence, and the developmental pattern of insects on buried bodies is important evidence for estimating the time of burial [20,21,22]. However, in practical case applications, it is necessary to consider whether insect colonization has preceded burial.

Sinaha [14] studied the life history and larval development patterns of this species on the coastal fish *Panna microdon* (Bleeker), including the mating mode, mating time, larval production time, and larval production quantity of this species, and described the morphology of larvae at different developmental stages. Currently, the studies on *S. princeps* have primarily focused on its molecular identification and the systematic classification [23,24]. However, there are a lack of systematic developmental studies of this species under constant temperatures in laboratory-controlled conditions, resulting in a lack of basic data for PMI_min_ estimation based on this species.

In this study, we investigated the development of *S. princeps* under seven constant temperatures (16–34 °C) in the laboratory, analyzing parameters such as developmental duration, larval body length, and pupal characteristics (such as pupal length, pupal width, and pupal weight), and establishing various developmental models at the above temperature. This study aims to provide foundational data for estimating PMI_min_ using *S. princeps* by establishing and studying laboratory populations collected from buried bodies, with a particular focus on buried remains.

## 2. Materials and Methods

### 2.1. Laboratory Population Establishment

This colony of *S. princeps* originated from a field experiment designed to study succession on carcasses buried 30 cm deep. Thirty rabbit carcasses were used in the study on succession of buried bodies in Suzhou City, Yangtze River Delta, during autumn of 2022. The protocols for the animal studies were approved by the Animal Protection and Use Committee and were conducted in accordance with the Soochow University regulations on animal experiments (ECSU-20190000109). Larvae of *S. princeps* were first detected on the fifth day post-burial, returned to the laboratory and were placed in a Petri dish (12 cm diameter) with 20 g of pork slices. The Petri dish was then placed in a rearing box with 2 cm of wet soil at the bottom. The rearing box was then placed into a microenvironment incubator (LHP-300H, Yingmin Co., Ltd., Changzhou, China) set at 28 °C to rear the larvae, with humidity at 70%, and photoperiod L12:D12. Pork was regularly replenished until pupariation was observed. After eclosion, the adults were placed in 90 × 90 × 90 cm rearing cages equipped with a 1:1 ratio of powdered milk and sugar for nourishment, and moistened sponges with fresh water for drinking. Fresh pork was provided in the rearing cages for protein supplementation after 5 days. The larvae were reared as described above. After five generations, experiments were started when the adult population reached 2000. The flies were identified via morphological methods [15,16] and supplemented with molecular identification using the *COI* gene. The corresponding gene sequence was submitted to GenBank (Accession Number: OQ860090).

### 2.2. Observation of Developmental Duration and Measurement of Larval Body Length

The developmental duration and larval length measurement experiments were conducted under laboratory conditions of 16, 19, 22, 25, 28, 31 and 34 °C, respectively. Twenty grams of fresh pork was placed in a Petri dish, which was then placed in an insect cage to trap adult flies for larviposition. Larviposition was monitored, and all larvae deposited within a 3 h window were collected and used for the experiment. The 25, 28, 31 and 34 °C experiments required that the number of larvae be more than 300, and 22, 19, and 16 °C experiments required that the number of larvae be more than 500. The time larvae were placed in the incubator was designated as point 0 for larval sampling, i.e., the 1st sampling. Thereafter, samples were taken at 4 h intervals until pupariation. Five larvae were collected from each sampling time point, and placed in hot water at 90 °C for 30 s then stored in 80% ethanol. The instar was identified using Zeiss 2000-C stereomicroscope (ZEISS, Oberkochen, Germany). The time of wandering, pupariation, and eclosion was recorded every 4 h throughout the experiment. The body lengths were measured with a digital vernier caliper (Sangon, Shanghai, China). Experiments were repeated three times at each temperature.

### 2.3. Measurement of Pupal Length, Pupal Width, and Pupal Weight

The same method as described in Section 2.2 was used to obtain larvae. Larvae (>200) collected within a 2 h window were reared as described above until wandering. Observations were then made 2 h until the pupae were formed and collected. If >50 pupae were collected in 2 h, twenty pupae were selected for weight, body width, and body length measurements. Pupal weight was measured with an electronic balance (FA1204N, Xingyun, Changzhou, China) with an accuracy of 0.0001 g. Pupal body length and width were measured with a digital vernier caliper (Sangon, Shanghai, China) with an accuracy of 0.01 mm. The above experiments were repeated three times at each temperature.

### 2.4. Data Analysis

A scatter diagram was utilized to establish an isomorphen diagram based on the mean value and standard deviation of the time required for five developmental events at seven constant temperatures, with the time from larviposition (h) on the X-axis and temperature (°C) on the Y-axis. An isomegalen diagram of *S. princeps* was plotted using development time as the X-axis, development temperature as the Y-axis, and body length change as the Z-axis. The above data analysis was conducted using Origin 2023. Python 3.9 was utilized to simulate the relationship between larval body length (mm) and time post-larviposition (h) using a modified logistic equation by Gao et al. [25] to generate a function for estimating PMI_min_.

Thermobiological parameters for each developmental stage of *S. princeps* were evaluated using the nonlinear thermodynamic model (Optim SSI), which was previously proposed by Ikemoto and Kiritani [26]. The SSI model is expressed as follows:

$$r(T)=\frac{\rho_{\emptyset }\frac{T}{T_{\emptyset }}exp[\frac{\Delta H_{A}}{R}(\frac{1}{T_{\emptyset }}-\frac{1}{T})]}{1+exp[\frac{\Delta H_{L}}{R}(\frac{1}{T_{L}}-\frac{1}{T})]+exp[\frac{\Delta H_{H}}{R}(\frac{1}{T_{H}}-\frac{1}{T})]}$$

where *r* is the Mean development rate (1/day); *T* is the Absolute temperature (K) (273.15 K = 0 °C); *R* is a Gas constant (1.987 cal/deg/mol); *ΔH_A_* is the Enthalpy of activation of the reaction that is catalyzed by the enzyme (cal/mol); *ΔH_L_* is the Change in enthalpy associated with low temperature inactivation of the enzyme (cal/mol); *ΔH_H_* is the Change in enthalpy associated with high-temperature inactivation of the enzyme (cal/mol); *T_L_* is the Temperature at which the enzyme is active at 50% because of low temperature (K); *T_H_* is the Temperature at which the enzyme is 50% active because of high temperature (K); *T_Φ_* is the Intrinsic optimum temperature at which the probability of the enzyme being in the active state is maximal (K); *ρ_Φ_* is the Development rate at the intrinsic optimum temperature *T_Φ_* (1/day) assuming no enzyme inactivation.

The above data analysis was conducted using Origin 2023 and R 4.3.0 (https://www.r-project.org/). Box plots of pupal weight, and 2Ys box plots of pupal body length and width were plotted using Origin 2023.

## 3. Results

### 3.1. Developmental Duration and Isomorphen Diagram

**Figure 1:**
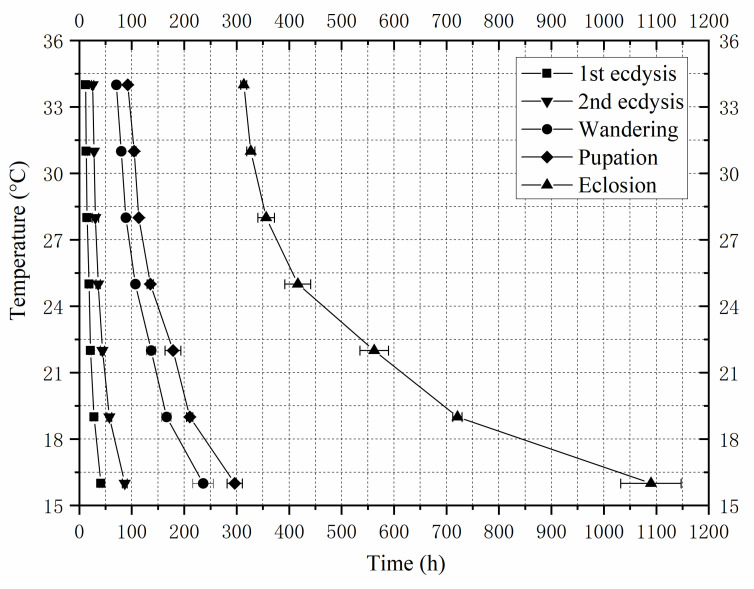
Isomorphen diagram of *Sarcophaga princeps*. The duration of each development milestone (first-ecdysis, second-ecdysis, wandering, pupariation, and eclosion) is plotted against the time from larviposition to the onset of each milestone. Each curve corresponds to a developmental milestone, and the error bar is the standard deviation of each milestone.

*Sarcophaga princeps* developed from larvae to adult in the laboratory at a constant temperature of 16–34 °C. The duration of each developmental event gradually decreased with increasing temperature. The total developmental time ranged from 1090.00 h at 16 °C to 313.67 h at 34 °C (Table 1). An isomorphen diagram was generated, showing different developmental events as different lines (Figure 1). Notably, the temperature and developmental time of *S. princeps* showed a negative correlation in all five developmental events. The intra-puparial period was longer than that of blowflies, accounting for 67.52–72.81% of the total developmental duration. By determining the on-site temperature (Y-axis) and the developmental stage of the flies, one can estimate the developmental time for that specific stage (X-axis).

### 3.2. Nonlinear Thermodynamic Optim SSI Model

**Figure 2:**
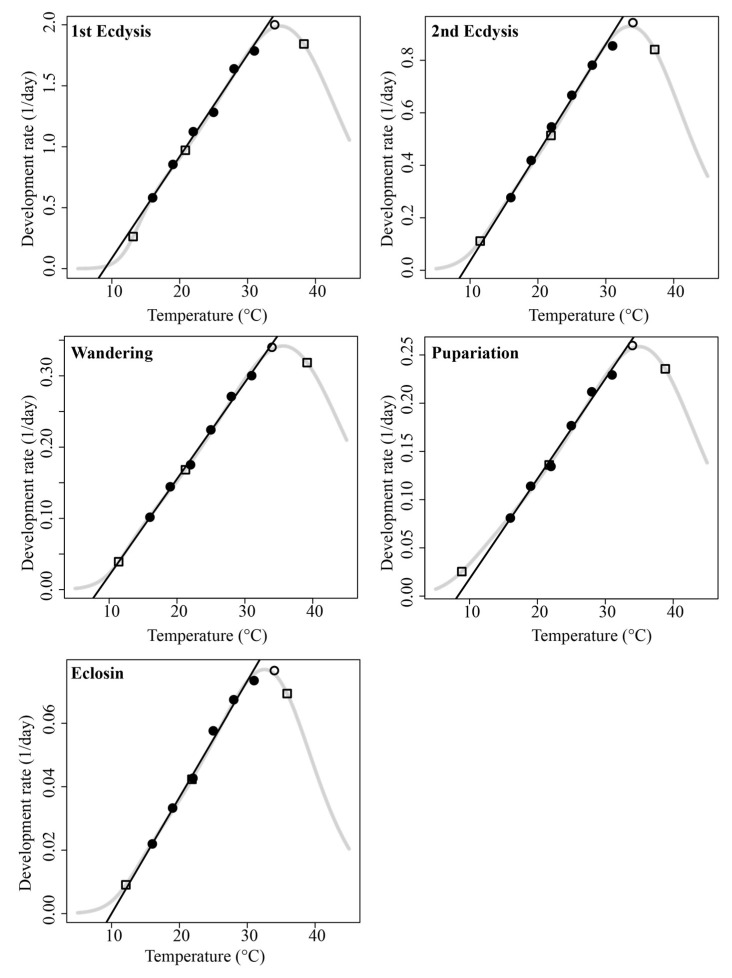
Nonlinear thermodynamic Optim SSI model for five developmental events of *Sarcophaga princeps*. Circles (black and white) indicate data points, and curves indicate the developmental rates predicted by the Optim SSI model. The three squares denote the predicted mean developmental rates of *T_L_*, *T_Φ_*, and *T_H_*. The black lines are generated by linear fitting of the black circle data, whereas the white circles are the data excluded from the linear fitting.

The nonlinear accumulated temperature model (Optim SSI) describing the relationship between the developmental rate of *S*. *princeps* and temperature across the seven experimental temperatures is shown in Figure 2. The figure shows that the developmental rate at 34 °C deviates from the linear fit, indicating that the nonlinear model provides a better fit and improves the accuracy of thermal response predictions. The thermodynamic parameters for each developmental event are shown in Table 2. The *R^2^* of each model is greater than 0.99, which indicated that the model simulation effect was better. Among the estimated parameters, three are of particular relevance to forensic entomology. The intrinsic optimum temperature (*T_Φ_*) varied little across different developmental stages, whereas the lower (*T_L_*) and upper (*T_H_*) critical thermal thresholds showed greater variation. These differences are likely attributable to varying adaptive capacities of the insects at different life stages.

### 3.3. Larval Body Length Variation and Isomegalen Diagram

**Figure 3:**
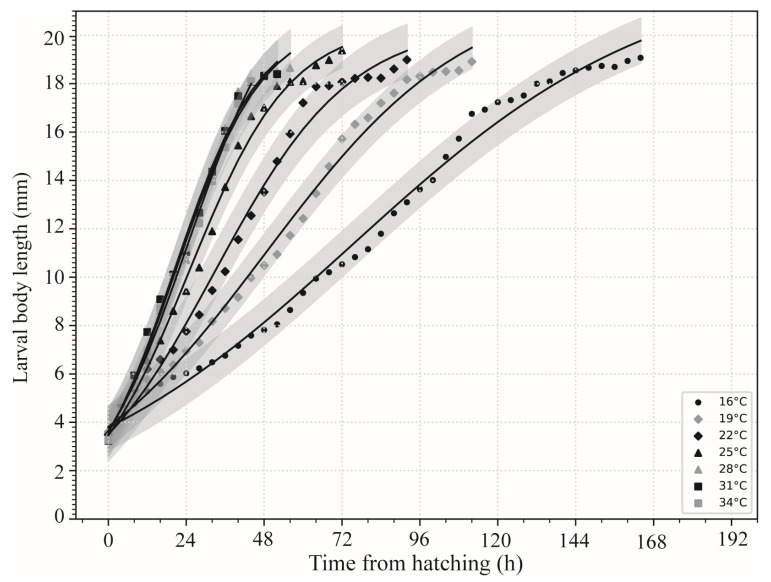
Larval body length variation over time in *Sarcophaga princeps* at different constant temperatures (16–34 °C).

**Figure 4:**
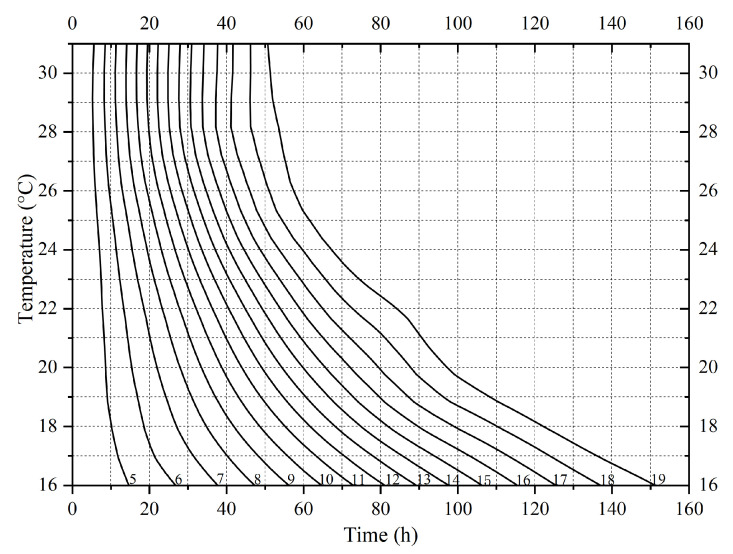
Isomegalen diagram of *Sarcophaga princeps*. Each line represents the length (mm) of developing larvae, with lengths ranging from 5 to 19 mm. The number at the bottom right of each line denotes the corresponding larval length.

The variation in larval body length of *S. princeps* over time at different constant temperatures is shown in Figure 3. Overall, the growth in larval body length followed a sigmoidal (“S”-shaped) pattern. Within the temperature range of 16–34 °C, the larval development rate increased with temperature. The logistic equations for the relationship between larval body length change and time since larviposition are shown in Table 3. The isomegalen diagram of *S. princeps* is shown in Figure 4. Numbers on the curves represent larval body lengths, with the lengths starting from 5 mm and increasing in 1 mm increments up to a maximum average of 19 mm.

### 3.4. Pupal Weight, Pupal Length and Pupal Width at Different Constant Temperature

**Figure 5:**
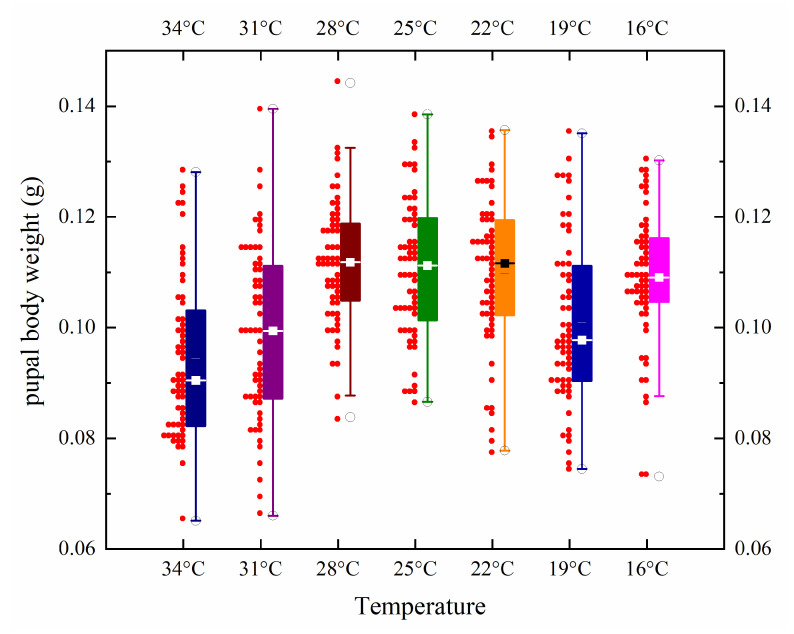
Box plots of pupal weight for *Sarcophaga princeps* pupae formed within a 2 h period at different temperatures, where “○” represents maximum and minimum values, “–” represents mean values, and “▪” represents median values.

**Figure 6:**
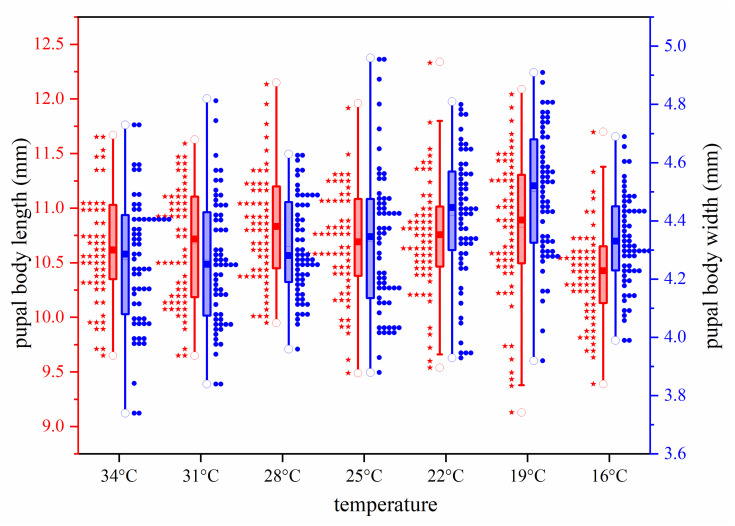
Box plots of pupal length and width for *Sarcophaga princeps* pupae formed within a 2 h period at different temperatures, where “○” represents maximum and minimum values, “–” represents mean values, and “▪” represents median values. The red five-pointed star on the left represents pupal length, and the blue solid circle on the right represents pupal width.

The maximum, minimum, and average values of pupal body weight, length, and width of *S. princeps* under seven constant temperature conditions are presented in Table 4. Among different temperature conditions, the maximum mean body weight is 0.1117 g, the maximum mean body length is 10.84 mm, and the maximum mean body width is 4.50 mm. For illustration, the pupal body weights, lengths, and widths of *S. princeps* at different temperatures are shown in Figure 5 and Figure 6. The results indicate that pupal weights, lengths, and widths are generally smaller at higher and lower temperatures, while the pupal weights obtained at 22–28 °C are larger, and the lengths and widths are larger at 19–25 °C.

## 4. Discussion

*Sarcophaga princeps* can colonize and reproduce on both buried and indoor cadavers, and belongs to the initial colonizing insect assemblage. In this study, we examined the developmental duration and larval body length variations of *S. princeps* across seven constant temperatures (16–34 °C). We established multiple models for estimating the PMI_min_, including isomorphen diagrams, the Optim SSI model, larval body length variation models, and isomegalen diagrams. These models can be used to estimate the age of *S. princeps*, providing valuable baseline data for forensic practice when this species is encountered at crime scenes.

To date, only one study has documented the life cycle of *S. princeps* [14]. However, the specific rearing temperature was not specified in that study, precluding direct comparison with our results. Another congeneric species, *S. peregrina*, exhibits comparable developmental periods at low temperatures (16–25 °C) but shorter periods at high temperatures (28–34 °C). The developmental periods of *Sarcophaga dux* at 15, 20, 25, and 30 °C were 1090.3, 566.6, 404.6, and 280.3 h, respectively, whereas our study found those of *S. princeps* at 16, 19, 25, and 31 °C to be 1090.00, 721.00, 416.67, and 327.00 h, respectively [9]. At comparable temperatures, *S. dux* exhibited a longer developmental period than *S. princeps* at 16, 19, and 22 °C but a shorter period at higher temperatures [27]. These data show that developmental duration varies even among congeneric species, a pattern that could be attributed to interspecific differences in native microhabitats, resource utilization, or physiological thresholds. Our findings underscore the risk of using congeneric data for PMI_min_ estimation and reinforce the necessity of species-specific developmental data [5,28].

Flesh flies, including *S. princeps*, are among the primary colonizers of cadavers, similar to blow flies [29,30,31]. However, in forensic entomology, research on the developmental patterns of blow flies far surpasses that on flesh flies, primarily due to the focus of most succession studies on exposed cadavers [7,31], where blow flies outnumber flesh flies in colonizing exposed remains. Another reason Sarcophagidae are more frequently neglected is that identifying their specimens, both adults and larvae, is more difficult than identifying specimens of Calliphoridae. In certain specialized environments, sarcophagid flies can act as the primary colonizers of cadavers and thus serve as critical entomological evidence for PMI_min_ estimation [10,32]. The *S*. *princeps* population used in this study was collected from our burial succession experiment conducted in the Yangtze River Delta region. In this experiment, calliphorid flies were unable to colonize the 30 cm deep buried rabbit carcasses, whereas *S. princeps* was the earliest to colonize and reproduce on these buried remains. Moreover, pupae of *S. princeps* were still detectable on the carcasses 120 days post-burial. This highlights the significant value of *S. princeps* in unique death scene environments (e.g., buried contexts). Compared to common blow fly species, sarcophagids typically exhibit longer developmental durations, enabling them to indicate longer PMI_min_. In the present study, the total developmental durations of *S. princeps* at 25 °C was 17.36 days (416.67 h), whereas that of calliphorid species such as *Calliphora vicina* [33], *C*. *megacephala* [34], *Chrysomya rufifacies* [35], and *Lucilia sericata* [36] was 16, 11.7, 12.1, and 15.5 days, respectively. This suggests that *S. princeps* requires a longer period to complete one generation on cadavers compared to these common calliphorid species.

The T_L_ is a key indicator reflecting the cold adaptability of sarcosaprophagous insects. In this study, the nonlinear thermodynamic Optim SSI model was used to calculate the *T_L_* of *S. princeps* as 11.11 °C, which is most similar to that of *S. peregrina* also collected from the Yangtze River Delta region [37]. The *T_L_* of *S. princeps* is greater than that of *Parasarcophaga similis* (9.60 °C) [38] and *Sarcophaga argyrostoma* (7.4 °C) [39] but lower than that of *S*. *dux* (12.26 °C) [27]. The *T_L_* of a species is primarily determined by its biological characteristics, though it may also be influenced by the calculation method as an estimated parameter. Previous studies have predominantly used the linear thermal summation model to calculate the *T_L_* of sarcosaprophagous insects [27,37,38,39]. However, in this study, we employed the nonlinear Optim SSI model proposed by Ikemoto and Kiritani [26], primarily because we observed a decline in the developmental rate of *S. princeps* at temperatures exceeding 31 °C, making the nonlinear model more suitable for data fitting to obtain more accurate estimates. In addition to *T_L_*, the nonlinear Optim SSI model can estimate the intrinsic optimum temperature (*T_Φ_*) and upper critical thermal threshold (*T_H_*), providing additional thermobiological insights into this species.

In this study, the maximum temperature tested was 34 °C, with no experiments conducted at higher temperatures. This is because our experiments demonstrated that the observed eclosion rate of *S. princeps* pupae at 34 °C was only 50.74 ± 2.54% (Table S1), leading us to infer that 34 °C is near the critical thermal threshold of this species. The T_H_ calculated by the nonlinear thermodynamic Optim SSI model was 35.88 °C, which largely supports this assumption. Shang et al. [9] also reported that *S. peregrina* cannot complete development at 35 °C, indicating that 34–35 °C is near the maximum developmental threshold for sarcophagid flies.

Numerous studies have noted that sarcophagid flies exhibit infrequent oviposition alongside their typical larviposition behavior [37,40,41], a phenomenon also observed in the present study. Specifically, when adult female *S. princeps* individuals were collected over consecutive days, they occasionally displayed oviposition behavior. However, the number of eggs laid was extremely low (generally fewer than 10), and these eggs either hatched immediately or failed to hatch, which is consistent with previous reports [37]. Overall, oviposition is infrequent in this species. Furthermore, considering that eggs hatch immediately upon deposition, we propose that this phenomenon can be disregarded in routine case extrapolations. Thus, when utilizing data from this study for PMI_min_ estimation in typical forensic cases, accounting for oviposition is unnecessary.

This study presents data on the growth and development of *S. princeps* under laboratory constant temperature conditions, and its growth and development here inevitably differ from that under natural fluctuating temperature conditions. Relevant studies have shown that *Protophormia terraenovae* [42] exhibits accelerated development under fluctuating temperatures, while species such as *C. megacephala* [43] and *S*. *peregrina* [44] show delayed development. Compared with laboratory constant temperature conditions, under natural fluctuating temperature conditions, the growth and development of *S. princeps* is also influenced by additional factors such as humidity, soil type, and interspecific interference. Future research should focus on two key areas: first, the specific impact of burial depth on the development of *S. princeps*; and second, the differences in its growth and development under natural fluctuating temperatures versus laboratory constant temperatures.

## Figures and Tables

**Table 1:** Average (±SD) duration (hour) to each development event from larviposition to the onset of each development event of *Sarcophaga princeps* at seven constant temperatures with 75% humidity and a light cycle of 12:12 (L:D).

Temperatures	First Ecdysis	Second Ecdysis	Wandering	Pupariation	Eclosion
16 °C	41.33 ± 2.31	86.67 ± 4.62	236.00 ± 19.52	296.33 ± 14.43	1090.00 ± 57.65
19 °C	28.00 ± 4.00	57.33 ± 4.62	166.33 ± 8.74	210.67 ± 6.11	721.00 ± 8.72
22 °C	21.33 ± 2.31	44.00 ± 4.00	137.00 ± 8.66	178.67 ± 15.28	562.33 ± 27.21
25 °C	18.67 ± 2.31	36.00 ± 4.00	107.00 ± 4.00	135.33 ± 5.86	416.67 ± 27.70
28 °C	14.67 ± 2.31	30.67 ± 6.11	88.67 ± 3.21	113.33 ± 2.89	356.33 ± 16.01
31 °C	13.33 ± 2.31	28.00 ± 0.00	80.00 ± 2.00	104.67 ± 4.16	327.00 ± 7.94
34 °C	12.00 ± 0.00	25.33 ± 2.31	70.67 ± 4.62	92.33 ± 2.52	313.67 ± 5.69

**Table 2:** Thermodynamic parameters of each developmental event of *Sarcophaga princeps* derived from the nonlinear Optim SSI model.

Parameter (Unit)	1st Ecdysis	2nd Ecdysis	Wandering	Pupariation	Eclosion
*T_Φ_* (℃)	20.83	21.91	21.25	21.73	21.85
*ρ_Φ_* (day^−1^)	0.99	0.53	0.17	0.14	0.04
*∆H_A_* (cal/mol)	1.31 × 10^4^	1.32 × 10^4^	1.27 × 10^4^	1.24 × 10^4^	1.44 × 10^4^
*∆H_L_* (cal/mol)	−1.14 × 10^5^	−7.26 × 10^4^	−8.15 × 10^4^	−5.75 × 10^5^	−7.87 × 10^4^
*∆H_H_* (cal/mol)	4.47 × 10^4^	4.94 × 10^4^	4.25 × 10^4^	4.53 × 10^4^	5.44 × 10^4^
*T_L_* (℃)	12.13	10.49	10.40	7.86	11.11
*T_H_* (℃)	38.3	37.20	39.17	38.83	35.88
*χ^2^*	8.20 × 10^−3^	5.21 × 10^−3^	6.03 × 10^−4^	1.01 × 10^−3^	2.82 × 10^−4^
*R^2^*	0.993	0.991	0.997	0.993	0.994

**Table 3:** A general model describing the relationship between the body length of *Sarcophaga princeps* larvae (L) (mm) and the time after larviposition (t) (hour).

Model	SE	
$L\left(t\right)=\frac{L_{m}}{1+\left(\frac{L_{m}}{L_{0}}-1\right)e^{-\lambda t}}$	/	(1)
$L_{0}\left(T\right)=4.4738-0.0371T$	±0.2600	(2a)
$L_{m}\left(T\right)=25.6924-0.1960T$	±0.8589	(2b)
$\lambda \left(T\right)=-0.0456+0.0042T$	±0.0025	(2c)

*L(t)* is the larval length at time t, *L_0_* is the initial length (i.e., at t = 0), *L_m_* is the final length, and *λ* is a parameter expressing growth rate. Equations (2a)–(2c) are the relationship between temperature (T) and *L_0_*, *L_m_* and *λ*.

**Table 4:** The maximum, minimum, and average values of pupal weight, length, and width of *Sarcophaga princeps* under seven constant temperature conditions (16–34 °C) (n = 60).

Temperatures	Pupal Body Weight (g)			Pupal Body Length (mm)			Pupal Body Width (mm)		
	Max	Min	Avg ± SD	Max	Min	Avg ± SD	Max	Min	Avg ± SD
34 °C	0.1281	0.0651	0.0944 ± 0.0145	11.67	9.65	10.65 ± 0.1877	4.73	3.74	4.27 ± 0.2218
31 °C	0.1395	0.0660	0.0991 ± 0.0157	11.63	9.65	10.67 ± 0.5261	4.82	3.84	4.27 ± 0.2165
28 °C	0.1442	0.0838	0.1117 ± 0.0113	12.15	9.95	10.84 ± 0.4902	4.63	3.96	4.31 ± 0.1662
25 °C	0.1385	0.0866	0.1106 ± 0.0125	11.96	9.49	10.69 ± 0.4885	4.96	3.88	4.33 ± 0.2503
22 °C	0.1357	0.0778	0.1097 ± 0.0135	12.34	9.54	10.76 ± 0.5148	4.81	3.93	4.42 ± 0.2251
19 °C	0.1351	0.0745	0.1009 ± 0.0151	12.09	9.13	10.82 ± 0.6631	4.91	3.92	4.50 ± 0.2208
16 °C	0.1302	0.0731	0.1088 ± 0.0122	11.70	9.39	10.41 ± 0.4312	4.69	3.99	4.33 ± 0.1608

## Data Availability

The original contributions presented in this study are included in the article/Supplementary Materials. Further inquiries can be directed to the corresponding authors.

## Supplementary Materials

The following supporting information can be downloaded at: https://www.mdpi.com/article/10.3390/insects16111153/s1, Table S1: developmental duration data of *S. princeps*. Table S2: larval body length data of *S. princeps*. Table S3: pupae data of *S. princeps*.
